# Application of the new 8th TNM staging system for non-small cell lung cancer: treated with curative concurrent chemoradiotherapy

**DOI:** 10.1186/s13014-017-0848-2

**Published:** 2017-07-21

**Authors:** Hoon Sik Choi, Bae Kwon Jeong, Hojin Jeong, Yun Hee Lee, In Bong Ha, Jin Ho Song, Ki Mun Kang

**Affiliations:** 1 Department of Radiation Oncology, Gyeongsang National University School of Medicine and Gyeongsang National University Changwon Hospital, 13 Samjungja-ro, Changwon, 51472 Republic of Korea; 2 Department of Radiation Oncology, Gyeongsang National University School of Medicine and Gyeongsang National University Hospital, 79 Gangnam-ro, Jinju, 52727 Republic of Korea; 3 Institute of Health Science, Gyeongsang National University, Jinju, Republic of Korea

**Keywords:** Non-small cell lung cancer, Staging, Chemoradiotherapy

## Abstract

**Background:**

The eighth tumor, node, metastasis (TNM) staging system (8-TNM) for non-small cell lung cancer (NSCLC) was newly released in 2015. This system had limitation because most patients included in the analysis were treated with surgery. Therefore, it might be difficult to reflect prognosis of patients treated with curative concurrent chemoradiotherapy (CCRT). Purpose of this study was to investigate clinical impact of the newly published 8-TNM compared to the current seventh TNM staging system (7-TNM) for locally advanced NSCLC patients treated with CCRT.

**Methods:**

New 8-TNM was applied to 64 patients with locally advanced NSCLC who were treated with CCRT from 2010 to 2015. Changes in T category and stage group by 8-TNM were recorded and patterns of change were evaluated. Survival was analyzed according to T category, N category, and stage group in each staging system, respectively.

**Results:**

Among the total of 64 patients, 38 (59.4%) patients showed change in T category while 22 (34.4%) patients showed change in stage group using 8-TNM compared to 7-TNM. Survival curves were significantly separated in the 8-TNM stage group (*p* = 0.001) than those in the 7-TNM (*p* > 0.05). Especially, survival of newly introduced stage IIIC by 8-TNM was significantly lower than that of others. On the other hand, there was no significant survival difference between T categories in each staging system.

**Conclusions:**

Subdivision of stage III into IIIA, IIIB, and IIIC by 8-TNM for patients treated with CCRT better reflected prognosis than 7-TNM. However, subdivision of T category according to tumor size in 8-TNM might be less significant.

## Background

Non-small cell lung cancer (NSCLC) that take the most part of lung cancer commonly use tumor, node, metastasis (TNM) staging system to make treatment decision and predict prognosis [1]. The current lung cancer staging system is the seventh TNM staging system (7-TNM). It was published by International Association for the Study of Lung Cancer (IASLC) in 2009 based on retrospective data of 81,496 patients [2–7]. 7-TNM acquired the new IASLC lymph node map and new definition of pleural invasion. However, it had limitation because original datasets were not designed to study TNM stage. In addition, not all descriptors of T, N, and M were validated.

The eighth TNM staging system (8-TNM) was released by IASLC in a revised form in 2015 [8–15]. The 8-TNM has analyzed a total of 77,156 patients, including 70,967 (92%) patients of NSCLC. This new staging system has major changes in T category, M category, and stage group. The T category is more subdivided by tumor size than that in the past staging system. Involvement of the main bronchus regardless of distance from the carina and development of atelectasis or obstructive pneumonitis regardless of range are now classified under the T2 category. Invasion to the diaphragm is changed from T3 to T4 category. The M category is subdivided under M1b for single extra-thoracic metastasis and M1c for multiple metastasis. In the stage group, former stage IA or IV is now subdivided under IA1, IA2, IA3 or, IVA and IVB, respectively. In addition, stage IIIC is newly added to stage III group. This new 8-TNM has advantages of having prospective trial and using certain algorithms that show the most distinct survival analysis. However, 8-TNM had limitation because most (85%) patients included in the analysis were operated, with surgery alone at 57.7%, chemotherapy plus surgery at 21.1%, radiotherapy plus surgery at 1.5%, and combined tri-modality at 4.4%.

For this reason, 8-TNM might well reflect the prognosis of early lung cancer patients treated with surgery. However, it might be difficult to reflect the prognosis of locally advanced NSCLC patients treated with concurrent chemoradiotherapy (CCRT) in approximately 70% of patients at the time of diagnosis [16]. Thus, the purpose of this study was to investigate change of stage and suitability of the revised 8-TNM to reflect the survival of those with locally advanced NSCLC treated with CCRT compared to 7-TNM.

## Methods

### Patients

Locally advanced NSCLC patients treated with curative CCRT at Gyeongsang National University Hospital between March 2010 and October 2015 were selected for analysis. This study was approved by Institutional Review Board (IRB) of Gyeongsang National University Hospital (IRB number: 2017–03-014). Inclusion criteria for this study were as follows: 1) histologically proven NSCLC, 2) Eastern Cooperative Oncology Group (ECOG) performance score between 0 and 2, and 3) treated with curative CCRT. Patients with 1) distant metastases, 2) ECOG performance score 3 or higher, 3) previous history of radiotherapy (RT), 4) other malignant disease, and 5) surgery of lung were excluded from analysis. Total number of patients treated with curative CCRT for NSCLC was 70. We excluded 6 patients who were lost to follow up. Therefore, a total of 64 patients were included for analysis. Patient characteristics (such as age, sex, smoking history, and performance status), tumor characteristics (such as tumor location, histology, tumor size, and number of metastatic lymph node), and treatment factors (such as CCRT, regimen of chemotherapy, and RT technique) were obtained for all patients.

### Stage

Physical examination, chest x-ray, pulmonary function test, chest computed tomography (CT), positron emission tomography-computed tomography, and brain magnetic resonance imaging were routinely performed for diagnosis and stage work up for all patients. For some patients, tissue diagnosis was performed through endobronchial ultrasound-guided transbronchial needle aspiration, transesophageal endoscopic ultrasound-guided fine needle aspiration or mediastinoscopy to confirm incorrect mediastinal lymph node metastasis. Stages were classified according to 7-TNM based on clinical stage. For the purpose of comparison, 7-TNM stages were reclassified and regrouped according to 8-TNM for each patient. Definition of T category and stage group for each staging system are shown in Tables 1 and 2.

**Table 1:** Definition of T category in 7-TNM and 8-TNM

	7-TNM	8-TNM
T1	Surrounded by visceral PL and not in MB	Surrounded by visceral PL and not in MB
T1mi	-	Minimal invasive adenocarcinoma
T1a	Size ≤2 cm	Size ≤1 cm
T1b	2 cm < size ≤3 cm	1 cm < size ≤2 cm
T1c	-	2 cm < size ≤3 cm
T2	MB (≥ 2 cm distal to carina), visceral PL, partial LA or OP	MB (regardless of distance from carina), visceral PL, part or all LA or OP
T2a	3 cm < size ≤5 cm	3 cm < size ≤4 cm
T2b	5 cm < size ≤7 cm	4 cm < size ≤5 cm
T3	Size >7 cm Parietal PL, chest wall, diaphragm, phrenic N, mediastinal PL, parietal pericardium, MB (< 2 cm distal to carina), entire LA or OP, separate nodule in same lobe	5 cm < size ≤7 cm Separate nodule in same lobe, parietal PL, chest wall, phrenic N, mediastinal PL, parietal pericardium
T4	Mediastinum, heart, great vessels, trachea, recurrent laryngeal N, esophagus, vertebral body, carina, separate nodule in a different ipsilateral lobe	Size >7 cm Separate nodule in a different ipsilateral lobe, diaphragm, mediastinum, heart, great vessels, trachea, recurrent laryngeal N, esophagus, vertebral body, carina

*7-TNM* seventh American Joint Committee on Cancer TNM staging system, *8-TNM* eighth American Joint Committee on Cancer TNM staging system, *PL* pleura, *MB* main bronchus, *LA* lung atelectasis, *OP* obstructive pneumonitis, *N* nerve

**Table 2:**
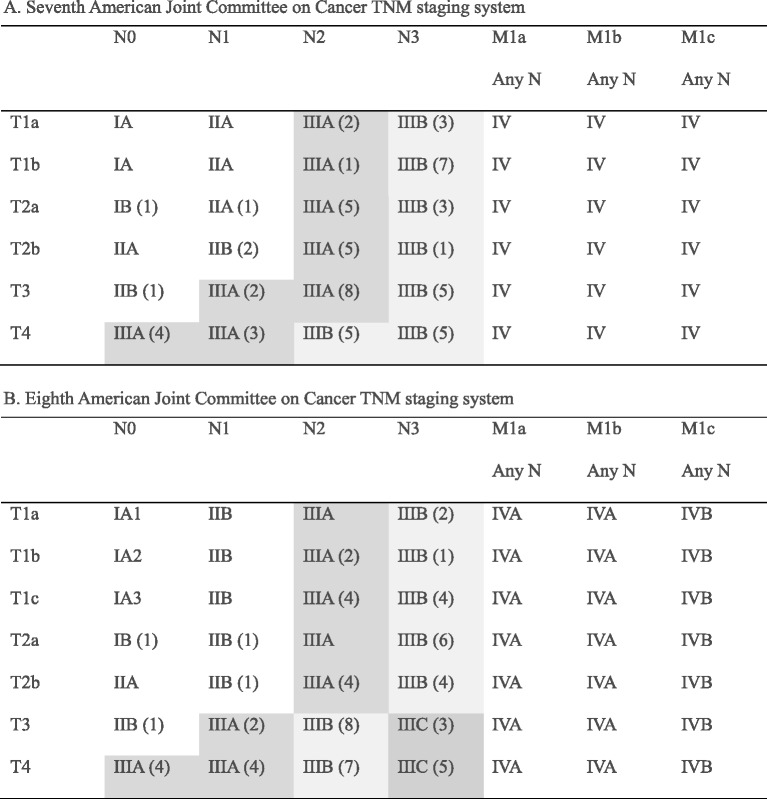
Stage grouping according to the staging system

Distribution of patients has been added in brackets

Stage group III was categorized using the following different colors: stage IIIA, gray; stage IIIB yellow; stage IIIC, blue

### Radiation therapy

All patients were immobilized with vac-lock in arm-up position and scanned CT images were obtained for lung at shallow normal breathing. These CT images were imported into Eclipse treatment planning system Version 10.0 (Varian Inc., Sunnyvale, CA, USA). Target and organ at risk (OAR) were delineated on those images. Gross tumor volume (GTV) was verified based on imaging data and pathologic data including tissue diagnosis for mediastinal lymph node. Clinical target volume (CTV) encompassed adjacent lymphatic station and ipsilateral hilum with a 10-mm margin from GTV. CTV was expanded uniformly by an additional 5-mm to generate planning target volumes (PTV) for tumor motion and set-up error. Total lung, spinal cord, and heart were delineated as OAR. Basically, three dimensional conformal radiotherapy (3D–CRT) plan was designed using 3-field. If the target volume was too extensive or too close to OAR, intensity modulated radiotherapy (IMRT) plan was created. All patients were prescribed a total dose of 60–66 Gy with 1.8–2 Gy per fraction. RT plan was normalized so that 100% of PTV received more than 95% of the prescribed dose. Lung was limited to mean lung dose ≤20 Gy with V_20Gy_ < 30%. Heart was limited to V_35Gy_ < 30%. Spinal cord was limited to maximum dose <45 Gy at any point.

### Statistical analysis

The overall survival duration was defined as the period from the date of end of RT to the date of any death. Disease-free survival duration was defined as the period from the date of end of RT to the date of any recurrence or death. Survival analysis was performed according to different T category, N category, and stage group in each staging system. Kaplan-Meier method was used for survival curves. Log-rank test was used to compare survival differences between staging systems. All analyses were performed using SPSS software (Version 21.0; SPSS, Inc., Chicago, IL, USA). *P* value <0.05 was considered statistically significant.

## Results

### Patients

A total of 64 patients were included in this study. Patient characteristics are listed in Table 3. Their median age was 69 years (range, 47 to 80 years). Most (92.2%) of these patients were males with squamous cell carcinoma (76.6%). All patients were treated with curative CCRT. Taxol based chemotherapy was used for most patients. Regarding RT technique, the percentages of patients subjected to 3D–CRT, IMRT, and mixed technique were 42.2%, 40.6%, and 17.2%, respectively. Prescription dose was 60 Gy for 40.6% of patients. It was 66 Gy for 59.4% of patients. The median follow-up duration in all patients was 24 months (range, 3 to 79 months), and the median follow-up duration in surviving patients was 40 months (range, 13 to 40 months).

**Table 3:** Patient characteristics

Characteristics		Number of patients (%)
Age (years)		Median: 68 (range 47–80)
Gender	Male	59 (92.2)
	Female	5 (7.8)
Smoker	No	18 (28.1)
	Past	34 (53.1)
	Current	12 (18.8)
Histology	SCC	49 (76.6)
	AD	13 (20.3)
	Others	2 (3.1)
ECOG performance status	0	8 (12.5)
	1	48 (75)
	2	8 (12.5)
CCRT	Taxol – cisplatin	36 (54.7)
	Taxol – cyclophosphamide	15 (23.4)
	Taxol – carboplatin	11 (17.2)
	Others	2 (3.1)
RT technique	3DCRT	27 (42.2)
	IMRT	26 (40.6)
	Mix	11 (17.2)

*SCC* squamous cell carcinoma, *AD* adenocarcinoma, *ECOG* Eastern Cooperative Oncology Group, *CCRT* concurrent chemoradiotherapy, *RT* radiotherapy, *3DCRT* three dimensional conformal radiotherapy, *IMRT* intensity modulated radiotherapy

### Changes of stage

According to 7-TNM T category, the number of patients with T1a, T1b, T2a, T2b, T3, and T4 was 5 (7.8%), 8 (12.5%), 10 (15.6%), 8 (12.5%), 16 (25%), and 17 (26.6%), respectively. According to 8-TNM, T category was reclassified. The number of patients with T1a, T1b, T1c, T2a, T2b, T3, and T4 was 2 (3.1%), 3 (4.7%), 8 (12.5%), 8 (12.5%), 9 (14.1%), 14 (21.9%), and 22 (31.3%), respectively. In total, T stage was changed in 38 (59.4%) patients. The predominant reason for these changes was due to the called tumor size was changed from 5-cm to 7-cm as T3 in 8-TNM instead of T2b in 7-TNM. Other reason was due to called tumor size over 7-cm as T4 in 8-TNM instead of T3 in 7-TNM.

Regarding stage group by 7-TNM, the number of patients with stage IB, IIA, IIB, IIIA, and IIIB was 1 (1.6%), 1 (1.6%), 3 (4.7%), 29 (45.3%), and 30 (46.9%), respectively. After regrouping stage group by 8-TNM, the number of patients with stage IB, IIB, IIIA, IIIB, and IIIC was 1 (1.6%), 3 (4.7%), 20 (31.3%), 32 (50%), and 8 (12.5%), respectively. A total of 22 (34.4%) patients had different stage groups. A total of 21 patients were up-staged (1 from IIA to IIB, 2 from IIB to IIIA, 10 from IIIA to IIIB, and 8 from IIIB to IIIC). One patient was down-staged from IIIA to IIB. Total change for stage III in the number patients was 29 to 20 in stage IIIA, 30 to 32 in IIIB, and 0 to 8 in IIIC. Distribution of patients according to the staging system is shown in Table 2. Changes in T category and stage group are shown in Table 4.

**Table 4:** Changes of T category and stage group in 7-TNM and 8-TNM

(A) T category									
		8-TNM							Total
		T1a	T1b	T1c	T2a	T2b	T3	T4	
7-TNM	T1a	2	3	0	0	0	0	0	5 (7.8%)
	T1b	0	0	3	4	1	0	0	8 (12.5%)
	T2a	0	0	4	3	3	0	0	10 (15.6%)
	T2b	0	0	0	0	1	7	0	8 (12.5%)
	T3	0	0	1	1	4	5	5	16 (25%)
	T4	0	0	0	0	0	2	15	17 (26.6%)
Total		2 (3.1%)	3 (4.7%)	8 (12.5%)	8 (12.5%)	9 (14.1%)	14 (21.9%)	20 (31.3%)	
(B) Stage group									
		8-TNM							Total
		IB	IIB		IIIA	IIIB	IIIC		
7-TNM	IB	1	0		0	0	0		1 (1.6%)
	IIA	0	1		0	0	0		1 (1.6%)
	IIB	0	1		2	0	0		3 (4.7%)
	IIIA	0	1		18	10	0		29 (45.3%)
	IIIB	0	0		0	22	8		30 (46.9%)
Total		1 (1.6%)	3 (4.7%)		20 (31.3%)	32 (50%)	8 (12.5%)		

*7-TNM* seventh American Joint Committee on Cancer TNM staging system, *8-TNM* eighth American Joint Committee on Cancer TNM staging system

### Survival

**Fig. 1:**
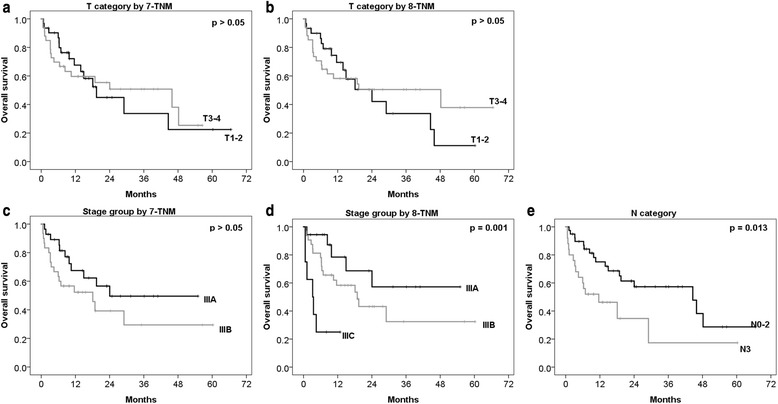
Overall survival curves according to T category, stage group, and N category in 7-TNM and 8-TNM. Curves of the T category by 7-TNM **a**, T category by 8-TNM **b**, stage group by 7-TNM **c**, stage group by 8-TNM **d**, and N category **e**. 7-TNM, seventh American Joint Committee on Cancer TNM staging system; 8-TNM, eighth American Joint Committee on Cancer TNM staging system

**Fig. 2:**
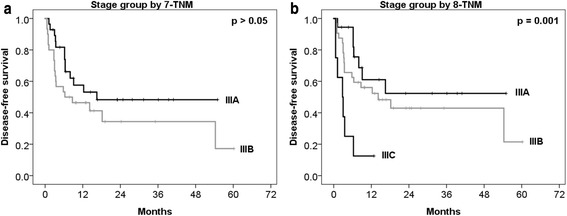
Disease-free survival curves according to stage group in 7-TNM and 8-TNM. Curves of the stage group by 7-TNM **a**, and stage group by 8-TNM **b**. 7-TNM, seventh American Joint Committee on Cancer TNM staging system; 8-TNM, eighth American Joint Committee on Cancer TNM staging system

Among these 64 patients, 28 (43.8%) patients died during the follow-up period. Accordingly, the 1 year, 2 year, and 3 year overall survival rates were 63.7%, 51.5%, and 44.7%, respectively. A total of 32 (50%) patients experienced some type of recurrence or death, and accordingly, the 1 year and 2 year disease-free survival rates were 49.8% and 41.6%, respectively. Overall survival was evaluated according to T category, N category and stage group in each staging system. Difference in overall survival according to T category was not significant in both staging systems. Differences in overall survival curves according to N category were statistically significant, although its definition in each staging system was the same. Especially, difference between N3 and other N categories was definitely significant (*p* = 0.013). Overall survival of stage group in 7-TNM showed different curves between stage IIIA and IIIB, although the difference was not statistically significant (*p* = 0.115). However, by 8-TNM stage group, the overall survival curves showed significant difference among stage IIIA, IIIB, and IIIC (*p* = 0.001). Especially, the difference between stages IIIB and IIIC was greater (*p* = 0.010) than that between stages IIIA and IIIB (*p* = 0.128). Overall survival curves for the T category, stage group, and N category according to 7-TNM or 8-TNM are shown in Fig. 1. With respect to disease-free survival, the stage group in each system was evaluated. Disease-free survival curves according to 7-TNM (*p* = 0.147) showed no significant difference, but based on 8-TNM (*p* = 0.001), differences in these curves were statistically significant. Disease-free survival curves for the stage group according to the 7-TNM or 8-TNM are shown in Fig. 2.

## Discussion

This study was planned under the assumption that 8-TNM might be difficult to reflect the prognosis of locally advanced NSCLC patients treated with CCRT due to limitation that most cases of IASLC were treated with surgery and only 4.7% cases were treated with CCRT.

The IASLC emphasized the effect of accurate tumor size, especially the prognosis of small sized tumor. In 8-TNM, T1 and T2 categories are more subdivided according to size compared to 7-TNM. Similarly, Okada et al. [17] have reported that survival is different according to small changes in tumor size when patients are treated with surgery. In contrast, David et al. [18] have reported that survival difference is significant for tumor size at around 3-cm. However, survival difference is not significant for other tumor sizes when patients are treated with radical RT. Our study for patients treated with curative CCRT showed no significant difference between subclass T1 and T2 categories, similar to radical RT results. This result indicates that subdivision of the T category might be less significant in patients treated with CCRT compared to surgery. The limitation of this interpretation is that there were little patients with early disease, survival of the T category confused by the N category, and there was lack of precision of tumor size measurement on CT images instead of the pathologic specimen. Therefore, further studies with more patients are needed to verify results of this study.

Several studies have reported heterogeneity of stage III and suggested the necessity of subdivision of stage III with various treatment strategies [19–22]. 8-TNM by IASLC subdivides stage III in 7-TNM into IIIA, IIIB, and IIIC. In our study, the number of patients of most heterogenous stage IIIA was reduced from 29 to 20 after subdividing stage III by 8-TNM. Several studies have reported about locally advanced lung cancer, patients of stage IIIB in N3 were excluded due to poor results and limitation of treatment [23, 24]. Stage IIIB with N3 in 7-TNM, meaning that T3 N3 and T4 N3 changes to stage IIIC by 8-TN3M. In our study, the survival curves between stages IIIA and IIIB were not statistically different according to the 7-TNM. This result was consistent with that in the study by Eaton et al. [25], which reported the survival results of the RTOG 0617 trial, and showed no difference in the overall survival between stage groups IIIA and IIIB according to the 7-TNM. In contrast, the survival curves for stages IIIA, IIIB, and IIIC according to the 8-TNM were well separated. The most distinctive difference was found between stages IIIB and IIIC. Based on this result, the N3 subcategory had the worst survival curve. This indicates that changes by 8-TNM might improve inhomogeneity of stage III and might well reflect prognosis of patients treated with CCRT. In addition, treatment strategies other than CCRT might be needed for patients at stage IIIC with relative poorly survival rate.

Regarding treatment methods for NSCLC according to stage, early stage NSCLC (stage I-II) is generally treated with surgery while advanced stage NSCLC (stage III–IV) is usually treated with CCRT. In this study, one patient was down-staged from stage IIIA to stage IIB based on the distance of tumor from carina. Therefore, when we only consider the stage for making a treatment decision, the treatment strategy could be different. Application of revised staging system might affect treatment strategies for some patients. Recently, novel treatment methods such as target agents, immunotherapy, and new RT techniques have been reported in cancer treatment field. Therefore, further prospective studies are needed to verify the new staging system when treated with novel treatment methods.

This study has some limitations due to its retrospective nature and small number of patients. Such limitations might have resulted in selection bias and influenced the power of statistical analysis. On the other hand, this study has the advantage of having homogeneous treatment data due to consistent methods of RT and chemotherapy performed by radiation oncologist and medical oncologist in a single institute.

## Conclusions

In conclusion, among changes in the new 8-TNM, subdivision of T1 and T2 categories might be less significant for locally advanced NSCLC patients treated by CCRT. However, subdivision of stage III to IIIA, IIIB, and IIIC is a clinically feasible change to reflect survival difference.

## Abbreviations

3D–CRT: three dimensional conformal radiotherapy
7-TNM: the seventh tumor, node, metastasis staging system
8-TNM: the eighth tumor, node, metastasis staging system
CCRT: curative concurrent chemoradiotherapy
CT: computed tomography
CTV: clinical target volume
GTV: gross tumor volume
IASLC: International Association for the Study of Lung Cancer
IMRT: intensity modulated radiotherapy
IRB: Institutional Review Board
NSCLC: non-small cell lung cancer
OAR: organ at risk
PTV: planning target volume
RT: radiotherapy
TNM: tumor, node, metastasis
